# SEM Investigation of Failure Mechanisms in Twaron^®^ Aramid Fibers Used for Personal Armors

**DOI:** 10.3390/polym17081058

**Published:** 2025-04-14

**Authors:** Alina Cantaragiu Ceoromila, Lorena Deleanu, Christian Popescu, Ionuț Lom, Alexandru Viorel Vasiliu, Petre Lucian Seiciu, George Ghiocel Ojoc, Simona Maria Sandu

**Affiliations:** 1 Cross-Border Faculty, “Dunarea de Jos” University, 111 Domneasca, 800201 Galati, Romania; alexandru.vasiliu@ugal.ro; 2 Center of Excellence Polymer Processing, “Dunarea de Jos” University, 111 Domneasca, 800201 Galati, Romania; 3 Center for Research and Innovation for CBRN Defense and Ecology, 225 Olteniței, 077160 Bucharest, Romania; chr_popescu@yahoo.com (C.P.); smsandu29@yahoo.com (S.M.S.); 4 National Institute for Aerospace Research (INCAS) “Elie Carafoli”, 220 Iuliu Maniu, 061126 Bucharest, Romania; lom.ionut@incas.ro; 5 Department of Machine Elements and Tribology, Faculty of Mechanical Engineering and Mechatronics, National University of Science and Technology “Politehnica”, 313 Splaiul Independenței, 060042 Bucharest, Romania; lucian.seiciu@as.info.ro; 6 Autonomous Flight Technologies, 1 Aeroportului, 077060 Clinceni, Romania; george.ojoc@gmail.com

**Keywords:** failure mechanism, aramid fibers, ballistic test, stabbing test, spike test, fibrillation, local bending, fracture, energy dissipation, scanning electron microscopy (SEM)

## Abstract

This paper presents a synthesis of failures of aramid fibers used in protective systems, with the help of SEM images obtained from three types of samples (panels made of fabrics with aramid fibers) tested against bullets, knives and spikes. This investigation is useful when using a step-by-step magnification and even macro photos in order to explain the mechanical failures of fibers. Several types of failure mechanisms (shear and tensile break, local bending, debonding from the matrix, fibrillation, local necking, etc.) were detected and discussed. Almost all of these failure mechanisms are present, with different densities of occurrence, in the studied panels made of aramid fibers. The description of failure mechanisms had to be conducted following the test conditions accurately. Failure mechanisms of aramid fibers are particularly relevant due to their specific molecular chains making them adequate for applications like ballistic and bladed weapon attacks.

## 1. Introduction

Aromatic polyamides are considered performance materials because they have very good mechanical and thermal properties [1,2,3], which are useful in high-tech industries (aeronautics [4], shipbuilding [5], applications at high temperature, military equipment [6,7]), but these fibers have huge potential in many other applications like the package and paper industry [8,9]. They have become advantageous substitutes for metals and ceramics, in many applications.

Commercially, the best-known aromatic polyamides are polyphenylene terephthalamide (PPPT) and poly mphenylene isophthalamide (PMPI). These fibers are added as woven or multi-axial directional fabrics and whiskers in structural composite materials, with different technologies and architectures, and have diverse applications: for stratified materials, coatings, as additives for advanced composites in the aeronautical and military industries, automotive, in leisure and sport industry, household goods, products for tribological applications, thermal protection articles, successfully replacing metallic materials and especially hazardous ones, like asbestos-based materials [10,11,12,13].

The high glass transition temperatures of aramids are near their decomposition temperatures, and their poor solubility in common organic solvents is a characteristic that induces processing difficulties, but recommends them in high-risk applications (fire-protection and military equipment) [14].

**Figure 1:**
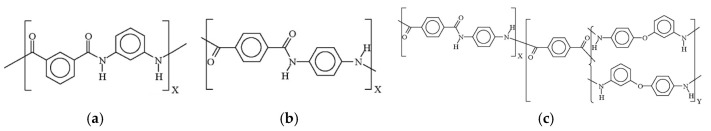
Molecular structure for aramid fibers [11]: (**a**) poly(m-phenylene isophthalamide (PMPI) (commercial grades: Nomex, Teijinconex [18,19]), (**b**) poly(p-phenylene terephthalamide PPPT (commercial grades: Kevlar [20,21,22,23,24], Twaron [25]), (**c**) co-poly-(p-phenylene/3,4’-oxydiphenilene terephthalamide (PPPT) (commercial grade: Technora [26]).

Chemists consider aromatic polyamides as synthetic polyamides, in which at least 85% of aramid groups are directly bonded to two aromatic rings. Figure 1 shows the structure of polyphenylene terephthalamide (PPPT) and poly mphenylene isophthalamide (PMPI), indicating several trade names.

The first aramid produced was poly(p-benzamide) (PPBA). PPBA was replaced by PPPT in the 1970s, under the now well-known name, Kevlar [15]. Their polycondensation was carried out at low temperatures, in a solution of terephthaloyl chloride (TPC) and p-phenylenediamine (PPD) in hexamethylphosphoramide (HMPA). Later, the solvent was successfully replaced by a N-methyl-2-pyrrolidone (NMP)/CaCl. PPPT is even less soluble than PPBA and can be converted into fibers from solutions in concentrated sulphuric acid, by spinning, at high temperatures [16,17].

The physical characteristics of aramid fibers (crystal lattice parameters, density, equilibrium moisture content, tensile properties at normal and elevated temperatures, thermal properties and chemical resistance) have been studied by [16,27,28,29].

García J.M. et al. [11] comparatively present the characteristics of PMPI and PPPT fibers and the values of mechanical characteristics are in the favor of PPPT. For PPPT, the tensile limit is much higher (2.9–3 GPa) and its Young modulus is 70–112 GPa as compared to 0.59–0.86 GPa and 7.9–12.1 GPa, respectively, for PMPI. The strain at break is almost ten times smaller for PPPT as compared to PMPI.

The use of aramid fibers is based on their very strong set of properties for efficient energy dissipation/absorption as well as improved damping properties at low vibrations, but also for noise attenuation.

Even if there are different solutions for individual protection (metallic plate, shear thickening fluids [30], ceramics for backing panels [31], 3D printing [32], etc.), composites with aramid fibers in a polymeric matrix or as prepegs are still the most effective and technologically readily available for resisting high-velocity impacts [10,33].

Failure mechanisms of aramid fibers are responsible for the energy absorption and the macro-scale behavior of a protective system [34,35,36].

The impact behavior of a stratified composite could be different, depending mainly on velocity (low or high). Low-velocity impacts are influenced by support conditions, high-velocity impacts induce local material damage, and boundary conditions are less influential on failure.

Woven fabrics have superior shear properties, but unidirectional (UD) fibers exhibit better characteristics in tensile loading. Hybrid panels, with both UD and woven fabrics, have been tested for ballistic protection, often with woven plies at the impact face [34,37,38].

Their failure mechanisms are of interest for researchers as these investigations could offer solutions to reduce, avoid or postpone the mechanical damage of these fibers. The authors of this paper focused on the failure mechanisms of Twaron^®^ aramid fibers used for high-risk applications like protection equipment against ballistic and bladed-weapon threats.

## 2. Structure of the Aramid Fiber

Aramid fibers, such as Kevlar [20] and Twaron [25], are widely used in bullet-proof vests and stab/spike protection due to their exceptional strength, high toughness and resistance to impact and penetration. However, they are subject to various failure mechanisms, depending on the nature of the threat (bullet impact vs. stab or spike attack) and environmental factors.

**Figure 2:**
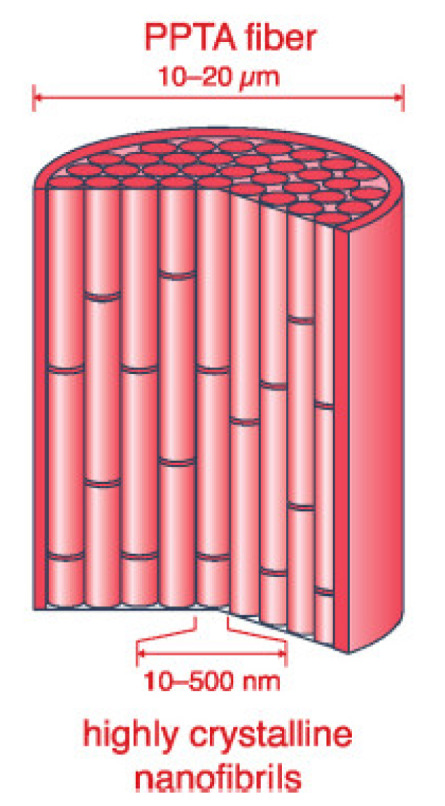
A model of the aramid fiber, proposed by [39].

In order to explain the failure mechanisms, the structure of aramid fibers has to be described and understood. Models for this outstanding fiber have been enhanced since the first commercially produced fiber, Kevlar, was introduced in the 1970s [16]. Figure 2 presents a simple model [39]; poly p-phenylene terephthalamide (PPTA) fibers are widely considered to consist of bundles of aligned crystalline nanofibrils, with high degrees of axial molecular orientation. They are separated in the lateral and axial directions by less ordered regions containing defects that may originate from chemical impurities or local concentrations of molecular chain ends and may be 20–25% of the total fiber volume [16,40,41,42].

The particularly good mechanical characteristics of aramid fibers rely on the polymer crystallinity and fiber fabrication by spinning. Fibers have to support loading from different directions and fiber-fiber and matrix-fiber contacts. The exact contribution of the single fiber behavior to the system performance is difficult to establish, but a statistical approach could explain the outstanding strength of their yarns [43]. Mechanical characterization of a single aramid fiber (its diameter being 9–12 μm) [44,45,46] is essential, even if the “translation” of this information to yarns, fabrics and final products (as vests) is difficult to carry out as scaling of fibers to larger systems prove not to have a reliably predictable nature.

We will discuss SEM images of aramid fibers using the fiber model presented by Richard C. et al. [47] that considered a multi-level microstructure. The molecular structure of the para-aramid monomer unit is linear, generating rigid rods. The typical molecular weights are around 40,000 g/mol, with an average chain length of 100–200 nm [48,49]. The spinning process generates a strong orientation and a high crystallinity of the aramid fibers. The resulting fiber has a complex multi-level structure with macromolecular chains aligned in the fiber length direction, connected by hydrogen bonds and van der Waals forces in sectional direction. The fiber structure consists of a core and a jacket. This core has a folded-sheet structure, with a typical dimension of 250 nm in the fiber length [42,50], that could be interpreted as crystallite [51].

The jacketcore structure, obtained by a melt–spinning process [51,52], suggests that the surface is uniformly and extremely oriented whereas the core is less perfectly packed and oriented. In the fiber core, the ends of the molecular chains tend to entangle, creating fewer resistant zones. Recent studies suggest the key role of fibrils as components of the fiber [52,53]. Fractured surfaces have been investigated with scanning electron microscopy and quite realistic failure scenarios have been proposed.

Kevlar^®^ fiber fracture is agreed to be “fragile” since failure occurs at relatively low strain levels (i.e., 2–3%) [54] which makes the observation even more difficult. In situ observations of failure mechanisms of an aramid fiber have been carried out in [55].

Under an optical microscope, fibrillation and fiber splitting were observed shortly before the final fracture of the fiber [56,57]. However, the optical microscopy resolution is too low for revealing failure details [57]. Other authors used scanning electron microscopy (SEM) coupled with a focused ion beam or a focused electron beam to notch fibers and induce fracture localization, estimating a fracture energy for Kevlar^®^ KM2 at around 1.1 kJ/m^2^ [58]. However, none of these authors have reported specifically on the mechanisms leading to final failure [59]. Fibrillation is also characteristic of some natural fibers under load [60].

**Figure 3:**
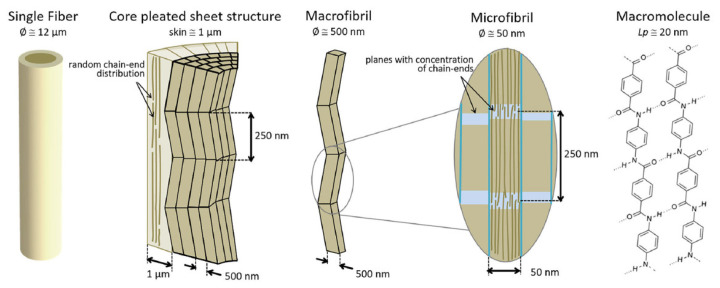
Multi-level architecture of para-aramid fiber, as proposed by Richard C. et al. in [47].

Figure 3 presents a multi-level architecture of a para-aramid fiber, explained by Richard C. et al. [47]. The fiber could have 9–12 μm. The structure is folded, with fibrils with different diameters, from tens of nanometers (named micro-fibrils) and 100–300 nanometers (named macro-fibrils).

The fiber jacket is around 1 μm thick. The macrofibrils contain several well-ordered “rods” with lengths of 250 nm, connected with smaller volumes containing entangled ends of the macromolecules. These volumes are prone to be damaged in traction or/and shear.

Tensile tests have been performed in the chamber of a scanning electron microscope in order to examine the change in the fiber microstructure during the test. The fiber was bonded to a frame with a 2 mm gauge length and then coated with a thickness of 3 nm of a gold–palladium alloy. Crack propagation was observed in situ on stretched fibers, from initiation to final failure. A low voltage of the electron beam was used (2 kV), focus was carried out far from the area of interest to avoid any electron damage and a very fast scanning velocity was selected [47].

The objective of this paper is to point out the failure mechanisms characteristic for the Twaron aramid fiber, used for personal protection (ballistic, stabbing and spike attacks). These were identified with a structure similar to those above-described but also with particular features due to the severe loading under ballistic knife and spike attacks.

## 3. Preparation of Samples Containing Aramid Fibers to Be Investigated Under Scanning Electron Microscopy

Preparing aramid fiber samples for scanning electron microscopy (SEM) involves several steps to ensure high-quality imaging. The authors present a step-by-step guide in Table 1. This could be applied, with differentiated aspects, to other polymeric fibers.

It is necessary to have samples that will fit inside the SEM enclosure [61]. The cutting of the sample should not disturb the area of interest. The maximum dimensions of the cut samples from the panels were 40 mm × 40 mm. This surface was enough to center the damaged area and to include boundary non-damaged areas. The fabrics made of aramid fibers are difficult to cut and the researcher had to pay attention not to pull out fibers or even yarns.

To investigate the sub-micron defects, it is necessary to provide magnifications of up to approximately ×50,000. However, during SEM analysis, the high level of surface roughness has to be taken into account; so, the exposed sample surface may not be uniformly metal coated. This could provide a poor resolution of fine details. Another limitation could be the thermionic electron source from the SEM structure, increasing acceleration voltage and electron damage to the area of interest.

In this study, the samples are golden coated with 6 nm gold alloy layer, with the help of Sputter Coater equipment (SPI Supplies, West Chester, PA, USA) and parameters of the coating device are vacuum pressure—6 mbar, plasma current intensity—around 18 mA and working distance—50 mm. The SEM images were obtained with the help of FEI Quanta 200 scanning electron microscope (from FEI Company, Hillsboro, OR, USA, now Thermo Fisher Scientific Inc., Waltham, MA, USA). Its characteristics include resolution—4 nm, magnification power—1 million times, software for command, analysis software with EDX spectrometer etc. (Genesis Spectrum software, version 3.5) [62]. Scanning electron microscopy (SEM) is successfully used to study composites [63], although the preparation of the investigated areas is relatively laborious, but it reveals specific failure mechanisms, especially at micro level.

## 4. Images of Initial (Non-Damaged) Twaron^®^ Aramid Fabrics and Fibers

Following the steps presented in Table 1 ensures accurate, high-resolution SEM images, allowing detailed analysis of the fiber morphology, surface structure and dimensions of the cross section of the fibers.

In this study, the observations on failure mechanisms of aramid fibers after ballistic, knife or spike impacts are carried out on the Twaron^®^ aramid fibers (fabrics and prepegs) are noted in Table 2, based on the producer’s catalogs [64,65].

**Figure 4:**
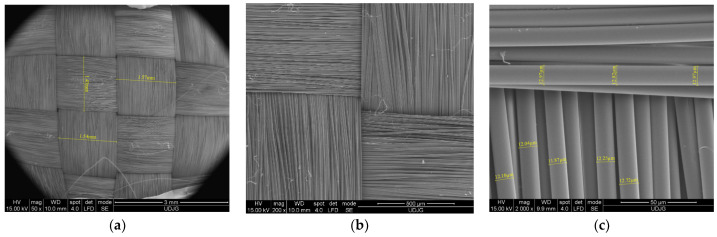
Different magnifications of Twaron^®^ CT736 fabric before being tested, in order to point out (**a**) yarn dimensions (×50), (**b**) the relative positions of fibers in a yarn, with weaving tightness (×200), and (**c**) fiber dimensions (×2000).

SEM images could be taken before the fibers were loaded in order to be compared to the damaged fibers. For instance, Figure 4 presents the fabric prepeg Twaron^®^ CT736 [65]: (a) at low magnification (×50), the tightness of the woven yarns and the yarn dimensions are visible; this is a required characteristic for withstanding ballistic and white weapon attacks; (b) at ×200, the fibers of the yarn are visible, with small changes in the fibers’ orientation; and (c) at ×2000, the fibers’ diameters were measured, revealing a narrow dispersion of their values, both on the same fiber (12.97–13.82 μm) and for several other fibers (11.87–13.82 μm); this reflects the quality of the fibers.

**Figure 5:**
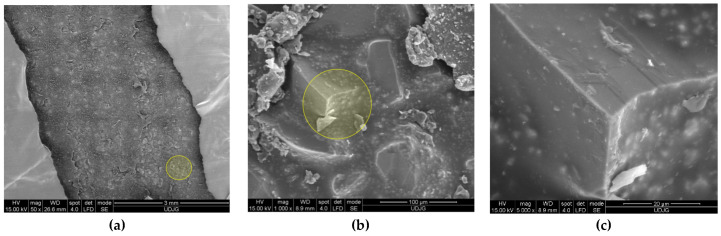
Face of the fabric Twaron^®^ SRM509, at different magnifications (yellow circles indicate the zoomed zones in the next image): (**a**) ×50, (**b**) ×1000, (**c**) ×5000.

Figure 5 presents SEM images of a prepeg Twaron^®^ SRM509, made of CT709 woven Twaron^®^ fabric + one-side silicon carbide coating, designated for stab and puncture protection, at different magnifications: (a) the coating, thin enough to view the pattern of plain woven fabric, at ×50 magnification; (b) at ×1000, the coating reveals the abrasive particles and the resin matrix that cover these particles, but several particles have their edges free of resin; (c) at ×5000, a single edge of a particle could scratch and abrade the knife or the spike, delaying its advance in the protective panel. This prepeg was used in panels with different layer numbers, in a research study that pointed out the influence of this parameter and also the influence of knife and spike energy [66,67].

**Figure 6:**
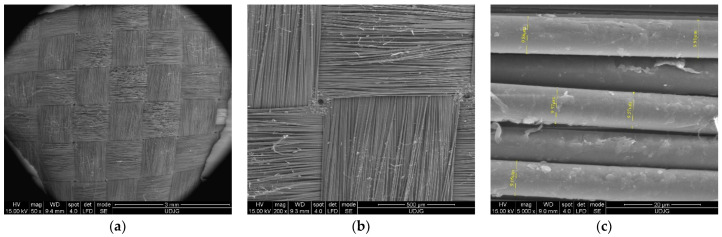
Different magnifications to underline the architecture of the back of the fabric Twaron^®^ SRM509: (**a**) ×50; (**b**) ×200; (**c**) ×5000.

Figure 6 presents the back of the prepeg Twaron^®^ SRM509: (a) a small magnification (×50) points out the tightness of the plain woven fabric and the yarn dimensions; (b) detail of the fibers’ arrangement in yarns and a technological detail; (c) at ×5000, the fiber diameter was measured and this dimension varies between 9.05 μm and 9.57 μm, at least in this SEM image; the fibers are coated with a very thin layer of polymeric coating.

## 5. Failure Mechanisms of Twaron^®^ Aramid Fibers in Systems for Individual Protection

### 5.1. SEM Analysis of Fibers Under Ballistic Hits

**Figure 7:**
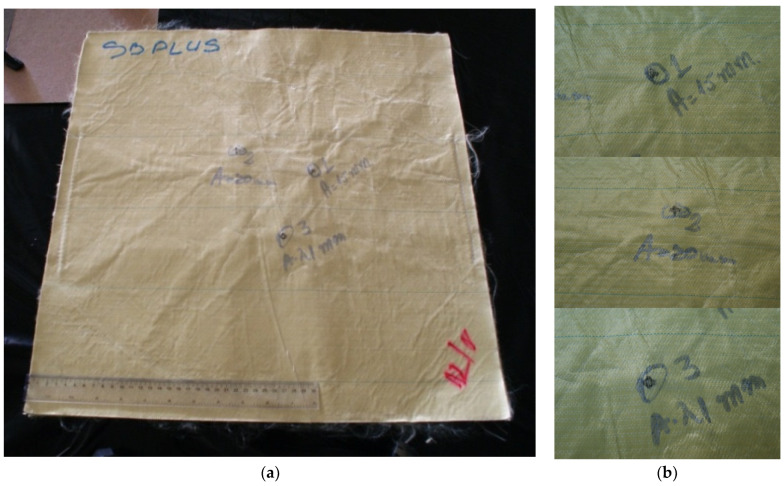

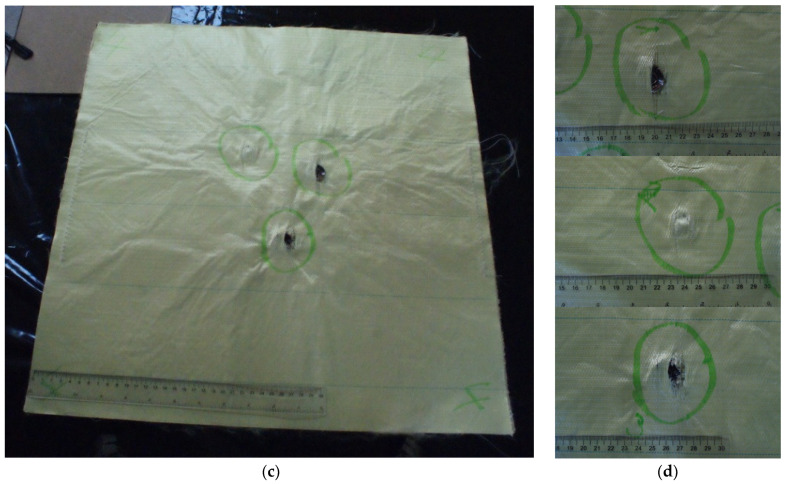
A tested panel made of 12 layers of LFT SB1plus, hit with a projectile 9 mm FMJ: (**a**) front view of layer 1 (notations are for the BFSs measured in the support material: A_1_ = 15 mm, A_2_ = 20 mm and A_3_ = 21 mm); (**b**) details of the damaged areas by the hits numbered in the order of their succession 1, 2 and 3; (**c**) front view of layer 4; (**d**) details of damaged areas 1, 2 and 3 [70].

Figure 7 presents the photos of two layers from a panel made of 12 layers of Twaron^®^ LFT SB1plus [64], after being hit with a 9 mm FMJ bullet (level IIA, NIJ Standard 0101.06 [68,69]), with partial penetration and a low average BFS (backface signature) of 19.44 m (average of BFS values for 18 hits on 6 panels, velocity impact range 410–430 m/s). Test conditions were as follows: air temperature: 21 ± 5 °C; relative humidity: approximately 70%; atmospheric pressure: 760 ± 15 mm Hg. The results of the ballistics tests on LFT SB1 and LFT SB1plus are presented in detail in [70,71]. Figure 6 presents the layers 1 and 4 of a tested panel made of 12 layers of LFT SB1plus, and details of the damaged areas of each hit. It is worth pointing out that the projectiles were stopped on the 5th layer. Figure 7d shows the position of bullet 1 and bullet 3. Bullet 2 was not entangled in the 4th damaged layer and fell when we cut the side stitched seams for taking the photos.

**Figure 8:**
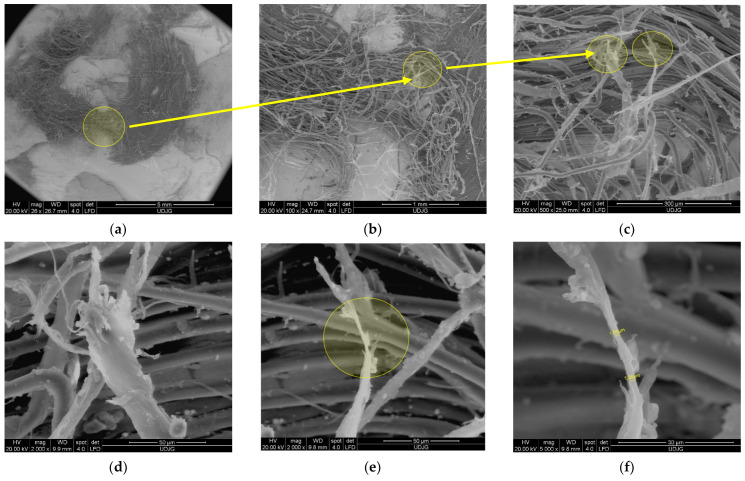
Different magnifications for understanding the failure mechanisms of aramid fibers that were trapped on the bullet: (**a**) view at low magnification (×26) of the fibers imprinted on the bullet nose; (**b**) a higher magnification (×100) of the image in the yellow circle in (**a**); (**c**) detailed image from the yellow circle in (**b**), at magnification of ×500; (**d**) broken fiber (×2000), detail of the left yellow circle in (**c**); (**e**) local thinning of a fibril, with different degrees of thinning (×2000), detail of the right yellow circle in (**c**); (**f**) detail of the fibril in the yellow circle in (**e**), at magnification of ×5000.

Figure 8 presents SEM images of the bullet nose, as extracted from a panel made of 12 layers of Twaron^®^ LFT SB1plus, at different magnifications of the same area of interest, in order to point out particular aramid fibers’ failures:

(a) at magnification ×26, there are visible short fragments of yarns embedded in the split jacket, but also in the lead core; this low magnification pointed out the copper jacket split as a flower (light grey color), lead core exposed (lighter milky color) and aramid fibers severely pressed on the bullet tip; all these emphasize the zone with maximum contact pressure: the fibers pressed and trapped on the jacket are broken because they are stretched during the bullet’s advance;
(b) a magnification of ×100 presents broken fibers, entangled and visibly stretched and broken in different ways;
(c) a magnification of ×500 presents fiber breaks after being loaded in traction (elongated, locally necked, local bending, local thinning); very small and isolated fragments of the matrix have remained on the fibers;
(d) a magnification of ×2000 revealed a shear cut of a fiber, another fiber with fibrillation and broken fibrils and a bent fiber (in the down-left corner);
(e) a fiber elongated and thinned; and
(f) at ×5000, fibrils could be measured (the central fibril has a thickness of 1.02–1.26 μm) and the shape of the failed fiber suggests successively breakage of the fibrils, meaning their break is delayed because of the fiber structure in fibrils.

Low magnification could also be obtained with a high-quality photo camera, as in [44], in order to evaluate the behavior of the stratified body armor.

**Figure 9:**
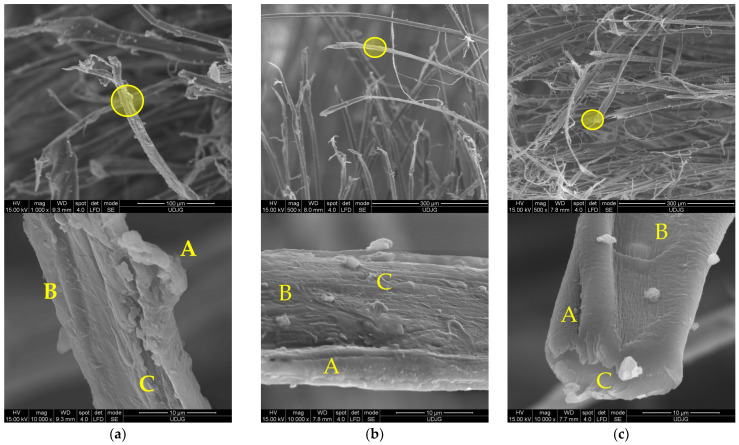
Structural evidence on SEM images for damaged aramid fibers: (**a**) fiber from first layer of a panel made of 5 layers of Twaron^®^ T730 WRT, hit by a 9 mm FMJ (v_0_ = 371 m/s): A—jacket fragment, partially detached from the core, a flake-like structure is visible, B—micro-fibrils with lateral smaller links, C—fibrillation; (**b**) fiber from the first layer of a panel made of 5 layers of Twaron^®^ T730 WRT, hit by a projectile.357 SIG (v_0_ = 448 m/s): A—local jacket split because of fibril separation, B—the disordered path, near the letter seems to be a band with entangled molecular ends, C—fibrils with straight alignment; (**c**) same layer as in (**b**), but another broken fiber: A—local fibrillation, with a fibril split in other three sub-fibrils, cut by shear, B—detaching of the micro short block in the zone of entangled ends of the macromolecules, C—broken surface of the fiber end revealing two levels of fibrils; milky droplets are matrix fragments still attached to the fiber.

Figure 9 presents high-magnification details of damaged aramid fibers which point out the structure described by Richard C. et al. [47]. The panels were tested at ballistic impact and at micro-scale, the qualitative difference between fibers from different panels is difficult to be evidenced. Test conditions were air temperature: 21 ± 3 °C, relative humidity: 47%, atmosphere pressure: 760 ± 15 mm Hg. All ballistics tests discussed in this paper used the Plasteline Rome 1 as backing material. The projectile velocities, mentioned in this paper, were measured with the help of the chronograph Oehler model 43. The target was set at 5 m in front of the nose of the ballistic barre, on a fire-arm table with a blow-back compensation.

Structural evidence on SEM images for a damaged aramid fiber is revealed in Figure 9 (the capital letters locate the discussed zones and the yellow circles from the first line of SEM images point out the zoomed areas, presented in the second line, at a magnification of ×10,000):

(a) a fiber from the first layer of a panel made of 5 layers of Twaron^®^ T730, hit by a projectile of 9 mm FMJ (v_0_ = 371 m/s): A—jacket fragment, partially detached from the core, a flake-like structure is visible, B—micro-fibrils with lateral smaller links, C—fibrillation;
(b) fiber from the first layer of a panel made of 5 layers of Twaron^®^ T730 WRT (specific mass 260 g/m^2^, Teijin Aramid, Arnhem, the Netherlands) [65], hit by a projectile of 357 SIG (v_0_ = 448 m/s): A—local jacket split because of fibril separation, B—the disordered path, near the letter seems to be a band with entangled molecular ends, C—fibrils with straight alignment;
(c) same layer as in (b), but another fiber; A—local fibrillation, with a fibril split in other three sub-fibrils, cut by shear, B—detaching of the micro short block in the zone of entangled ends of the macromolecules, C—broken surface of the fiber end revealing two levels of fibrils; milky droplets are matrix fragments still attached to the fiber.

Generally speaking, many of the discussed failure mechanisms are met in all three cases here analyzed, but they could have particular aspects, and different generated densities and scanning electron microscopy (SEM) could evidence them.

Based on our experience in using SEM for investigating structures of polymeric materials (composites [72,73] and polymeric blends [74]), we start by analyzing the largest area (10 mm × 10 mm) under the microscope, at very low magnification (×20) and we continue with step-by-step magnification, until the highest magnification possible to be carried out with the microscope (×10,000, ×20,000). This allows for describing failure scenarios and their realistic evaluation.

**Figure 10:**
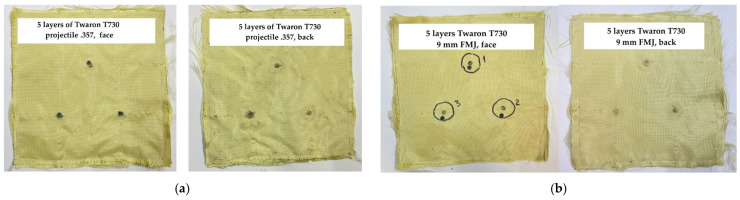
Panel made of 5 layers of Twaron^®^ T730 WRT: (**a**) panel face and back (three hits with .357 projectile, (**b**) panel face and back (three hits with 9 mm FMJ projectile). Test conditions were air temperature: 21 ± 3 °C, relative humidity: 47%, atmospheric pressure: 760 ± 15 mm Hg.

**Figure 11:**
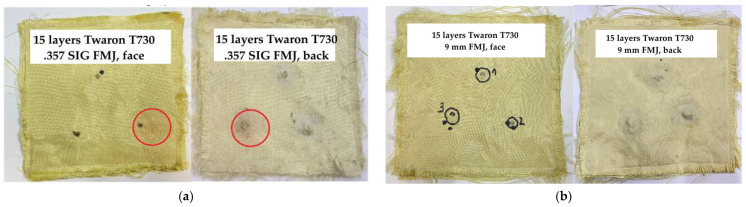
Panel made of 15 layers of Twaron^®^ T730 WRT: (**a**) panel face and back (three hits with .357 SIG FMJ projectile, the red circles show the hit with total penetration); (**b**) panel face and back (three hits with 9 mm FMJ projectile, with no total penetration for any of the three hits) (Test conditions were air temperature: 21 ± 3 °C, relative humidity: 47%, atmospheric pressure: 760 ± 15 mm Hg).

Figure 10 and Figure 11 present photos of tested panels made of layers of Twaron^®^ T730 WRT, squarely stitched at 8–10 mm from the edges, from a study with the objective of having a protection for different levels (and projectiles). Panels have dimensions 300 mm × 300 mm and the distance between hits is 1200 mm. The numbers 5 and 15 were selected as extremes for the layer number range. These photos show the tested samples from which the investigation started to explain failure mechanisms of aramid fibers.

The data obtained for the BFS acquired for the tested panels made of 15 layers of Twaron^®^ T730 WRT are shown in Table 3. One may notice that the threat of 9 mm FMJ produced a BFS smaller than the admissible one, of 44 mm as requested in [68]. Results for the projectile .357 SIG FMJ are not acceptable as there was a total penetration and the values of the other two hits are high enough to consider that this panel is not secure.

**Figure 12:**
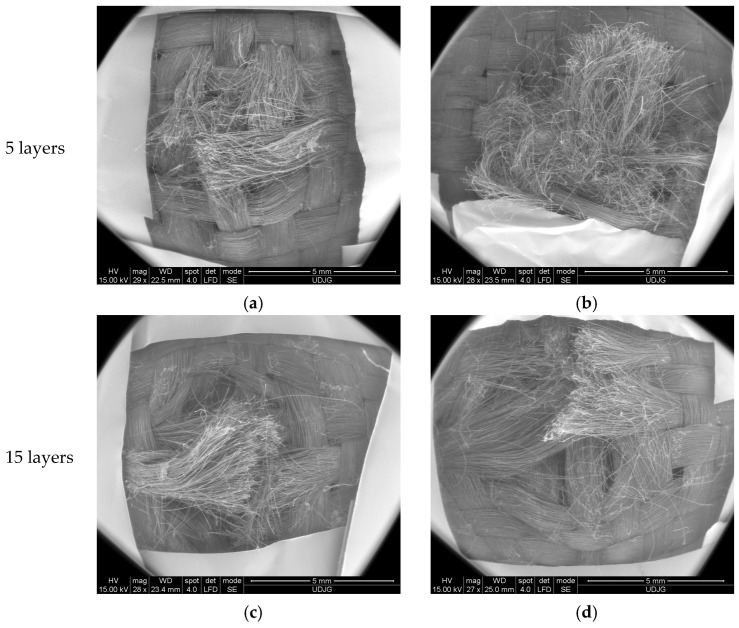
View of the orifice generated by bullet impact on the studied panels made of Twaron^®^ T730 WRT: (**a**) 9 mm FMJ, v_0_ = 372 m/s; (**b**) .357 SIG, v_0_ = 448 m/s; (**c**) 9 mm FMJ, v_0_ = 371 m/s; (**d**) .357 SIG FMJ, v_0_ = 447 m/s (Test conditions were air temperature: 21 ± 3 °C, relative humidity: 47%, atmospheric pressure: 760 ± 15 mm Hg).

High-velocity impact generates localized stress and strain on aramid fibers, causing the fiber breakage. Images in Figure 12 are taken at a low magnification in order to show how the same fabric behaves under different ballistic conditions. The first layer of each panel exhibits several broken yarns, but it is difficult to associate the image to different panels (5-layer panels have total penetration for both projectiles and 15-layer panels stop the bullets).

The flat nose of .357 SIG FMJ compresses yarns and its advance in the panel produces the fracture of the fibers, usually in one part of the yarn. The same scenario is for the round nose projectile 9 mm FMJ (full metal jacket). Thinner panels also have a disordered bunch of fibers, as the elasticity of the panel until the yarns are broken, allows for larger elastic deformations and after the passage of the bullet (total penetration), the fibers have a movement of whipping and this produces entangling of the fibers, which is more pronounced at higher velocities. This localized action of the projectile could also be evidenced by FEM analysis [70,75]. These panels were selected for having a full penetration (5-layer panel) and a partial one (32-layer panel) based on previews testing and armorer experience.

Key failure mechanisms of aramid fibers under ballistic impact include fiber fracture, fiber delamination from the matrix (if there is a matrix), fibers’ pull-out, plastic deformation with two distinct aspects, necking and flattening, bending and microbuckling.

High-velocity bullets create extreme localized strain and stress, causing fibers to break in many ways. This failure typically occurs through fracture mechanisms due to cut (shear) or traction. The aspect of the broken fibers is different, but both situations are met in ballistic impact on panels made of aramid fibers.

**Figure 13:**
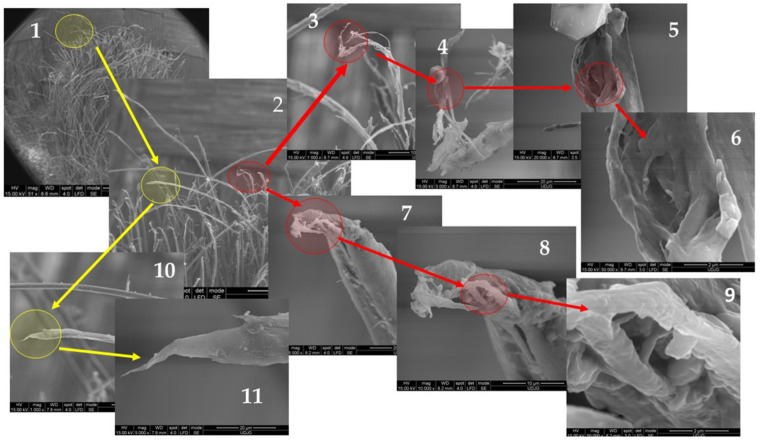
Step-by-step magnification for highlighting different failure mechanisms of aramid fibers, after ballistic impact, at different magnifications: 1—×50; 2—×200; 3—×1000; 4—×5000; 5—×20,000; 6—×50,000; 7—×5000; 8—×10,000; 9—×50,000; 10—×1000; 11—×5000. The circles and arrow paths indicate areas of interest and their sequence in increasing magnification.

All SEM images in Figure 13 are taken from the first layer of a panel made of 5 layers of Twaron^®^ T730 WRT fabric (Teijin, Arnhem, The Netherlands) after being hit by a projectile .357 SIG, with an impact velocity of 448 m/s. Figure 10 gives an example of how to point out different failure mechanisms of aramid fibers, starting from the yarn image.

The arrows in red and yellow indicate the next magnification of a field of interest. Thus, the SEM images in Figure 13 are detailed as

1. the SEM image with the lowest magnification (×50); it presents a main yarn (the yarn bearing the hit of the projectile and usually with the highest degree of damage), but the identification of breaking mechanisms of the fibers is not yet visible;
2. a magnification of the SEM image 1 (at ×200), where there are two damaged fibers to be discussed; the authors marked these two fibers with a red circle and a yellow one; these colors are retained in the following images if the fiber is the same as in the initial circle;
3. a detail of the broken fiber, with a thinning (necking) of the broken fiber end (×1000), showing broken fibrils, with different sizes that point out zones with differences in mechanical properties because of the discontinuity in the molecular order and size;
4. next magnification (×5000) shows how the fibrillation acts in this fiber, revealing non-uniform fibrillation and a droplet of resin (in the upper part of the fiber) remained stuck on a fibril;
5. detail of a local split among fibrils (×20,000);
6. a detail of the zone with fibrillation (×50,000);
7. a detail of the red circle in image 2, at higher magnification (×5000) that pointed out a shear cut with thinning on the fiber end, reflecting the high-velocity process (the ballistic impact);
8. a detail of the broken end of the fiber (×10,000);
9. very thin microfibrils are visible between fibrils, on the fiber direction (×50,000);
10. the image shows the fiber on about 100 microns length (×1000), showing many failure mechanisms: end break by tension, necking of the fiber, locally splitting of the fibrils;
11. the detail of broken fiber (×5000) suggests a sudden breakage in tension, with a short portion of the fibril with a conical chape, meaning yield of the polymer.

Even if these fibers have excellent thermal stability compared to many other polymeric fibers, prolonged exposure to high temperatures can lead to breaks in chemical bonds and weight loss [76]. Exposure to oxygen at high temperatures accelerates the degradation of aramid fibers. This process involves surface oxidation and chain scission. Oxygen reacts with the fiber’s surface, leading to discoloration and brittleness.

Certain environmental conditions can exacerbate mechanical failure, including moisture and UV radiation [77]. The effect could be synergic when both these factors act together. Mechanisms of chemical failure include hydrolysis and degradation by acids, alkali [74], oxygen and solvents.

Delamination could be noticed at micro level or at macro level: at first, this could be associated with the debonding of the fibers from their matrix. At mezzo or macro level, delamination is visible between layers. Multiple layers of woven aramid fabric are often used for vests. Impact forces can separate these layers, reducing their energy-absorbing capability.

In laminated aramid composites, cracks in the resin matrix occur, weakening the structure of the entire panel. The impact force may pull yarns from the structure, reducing the fabric’s ability to distribute the load. This damage is more visible with a smaller magnification of investigation in order to see several yarns and compare their positions after the impact; good-quality photos could point out this process [78].

Ballistic impacts generate heat, which may degrade fibers locally, especially if multiple impacts occur in a short time. For aramid fibers, this is less visible for impact velocities until 400–450 m/s, but for other fibers or tapes, as Tensylon [79], the thermal effect has to be taken into consideration because temperatures generated by the impact could soften or even locally melt the polymer, changing its behavior during the impact. The heat generated by impact softens these fibers, even melts them, but a beneficial aspect in arresting the projectile is that the friction coefficient increases and the soften/melted polymer adheres to the bullet and tends to absorb some of its energy.

**Table 3:** Experimental results for the two panels used for the SEM investigation.

Projectile	Hit Number	Measured Velocity (m/s)	Result	BFS (mm)
.357 SIG FMJ	1	445	PP	40
	2	447	PP	44
	3	447	PT *	-
9 mm FMJ	1	370	PP	36
	2	376	PP	35
	3	372	PP	36

* PT—total penetration, PP—partial penetration, BFS—backface signature [80].

Necking (as cross section change) and flattening are further critical failure mechanisms; first, particularly under tensile loading in aramid fibers, and second, under compressive loading.

An overview of how necking occurs and its role in the failure process now follows. It could be present in any of the analyzed loadings (projectile impact, knife hit or spike attack). Necking of aramid fibers refers to the localized reduction in the cross-sectional area that occurs when a material is subjected to tensile stress beyond its yield point. In aramid fibers, necking typically involves the alignment and stretching of polymer chains, which can lead to fibrillation and successively tear off the fibrils from their weaker end links, followed by catastrophic failure. This failure mechanism has several stages in time:

- an initial deformation: when a tensile load is applied, aramid fibers initially undergo elastic deformation,
- when load increases, plastic flow occurs, causing the molecular chains to start sliding past each other,
- generation of a localized contraction: beyond the yield point, deformation localizes in a small region, reducing the cross-sectional area (the neck formation), increasing stress concentration in that region,
- fracture propagation: as the necked region carries out most of the load, it becomes the point of ultimate failure, leading to fiber breakage.

**Figure 14:**
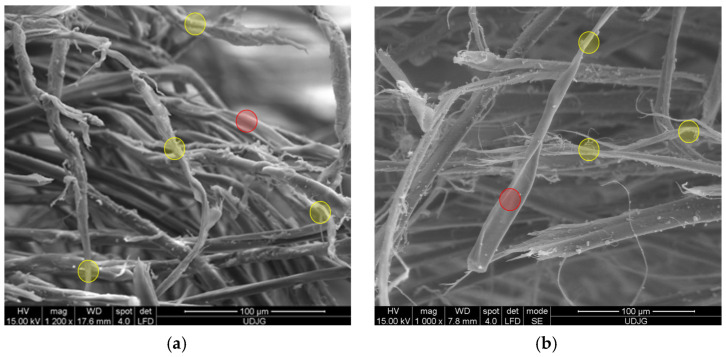
Fiber deformation: yellow circles—necking, red circles—flattening: (**a**) Twaron^®^ CT 709 with PVB matrix, 32 layers, after a hit with a 9 mm FMJ bullet [70], (**b**) front view of layer 1, from a panel of 15 layers of Twaron^®^ T730, hit by a projectile .357 SIG, v_0_ = 448 m/s.

Figure 14 presents fiber deformation in order to give examples of the above-discussed deformation, necking and flattening. Tests noted in Figure 14 could be considered level IIA [68,69], taking into account the projectile type and its impact velocity, v_0_.

Factors influencing the necking could be considered strain rate, temperature, defects or/and impurities within the fiber, fiber orientation. Higher strain rates (like those resulting from ballistic impacts) reduce the extent of necking since fibers may fracture before significant necking occurs. In stab and spike attacks, there is a high probability that this failure will occur for the fiber situated in the last layers of the panel as the fibers have to withstand larger deformation. Elevated temperatures can facilitate necking by increasing molecular mobility. Necking is more pronounced in uniaxial fibers. In woven fabrics, the fiber interaction within the weave can help distribute stress and delay necking. Any inconsistencies in the fiber structure can initiate necking at lower stresses.

Fibrillation refers to the splitting of aramid fibers into finer, thread-like fibrils, when subjected to mechanical stress. This is a common failure mode in high-performance fibers, with long linear molecular chains. This failure mechanism could be noticed both at the broken ends of fibers and localized on the fiber length.

Aramid fibers are composed of bundles of microfibrils aligned along the fiber axis. Under stress, these fibrils separate. When a load is applied, high-stress concentrators are created and the polymer matrix cracks and microfibrils fail progressively, creating fine, hair-like fibrils. Fibrillation dissipates energy across multiple fibrils, enhancing the material’s toughness. This mechanism helps delay complete failure. Fibrillation increases the energy required for complete fracture, contributing to the high resistance to impact of the aramid fibers.

The implication of fibrillation in energy absorption could be qualitatively differentiated in the three cases studied in this paper.

During a ballistic impact, fibrillation helps dissipate energy by creating multiple small fractures rather than a single catastrophic break.

### 5.2. SEM Analysis of Fibers Under Knife and Spike Attacks

**Figure 15:**
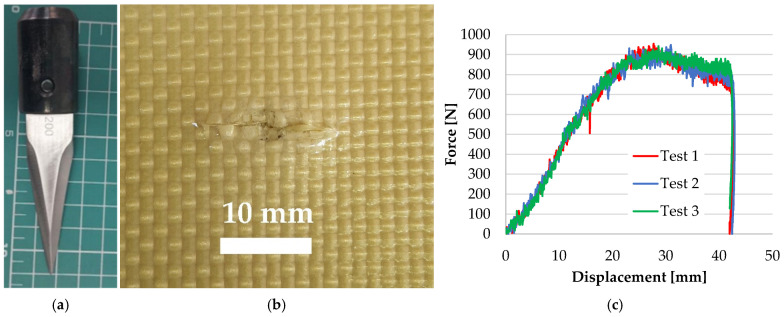
(**a**) Knife S1, with the blade geometry as in [80]; (**b**) the face of first layer from a panel of 16 layers of Twaron^®^ CT 736 CMP (thickness of 8.44 mm), after a test with a strike energy of 24 J (test carried out on INSTRON CEAST 9350 drop tower impact system, at INCAS Bucharest, Romania) [67]; (**c**) force–displacement curves for the three tests for the 16-layer panel of Twaron^®^ CT 736 CMP, under the same conditions (knife S1, 24 J).

The failure mechanisms when aramid fibers are hit by a knife (Figure 15a) are identified on the sample presented in Figure 15b. Environmental conditions for stab and spike tests, here presented, were air temperature: 19 ± 5 °C, relative humidity: 70%, atmospheric pressure: 764 ± 15 mm Hg. The backing support for stab and puncture tests were materials with equivalent mechanical characteristics as in [80], but tests presented in this paper were conducted on small samples (150 mm × 150 mm, only one hit), circularly fixed in the drop test device (Instron CEAST 9340, produced by Instron Norwood MA USA from INCAS, Bucharest, Romania). This test machine measures velocity, energy and force during the test (values of these parameters for ranges of energy and number of panel layers are given in [66,67]). Figure 15c presents the force–displacement curves for the three tests carried out under the same conditions and one may notice the very small differences among the shapes and values of these curves, showing a good repeatability for the tested samples and also noting that any of the tested samples could be considered typical for the investigated damaged areas.

**Figure 16:**
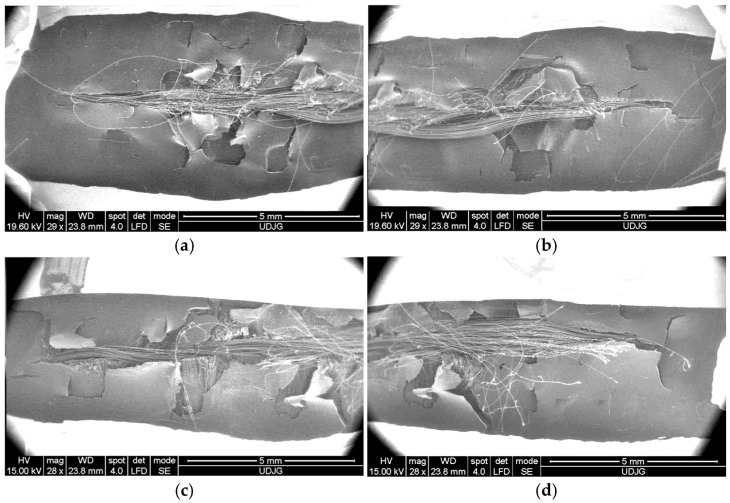
SEM images of the knife cut in the panel made of 16 layers of Twaron^®^ CT 736 CMP (thickness of 8.44 mm), after a test with a strike energy of 24 J: (**a**) left side of the cut on the impacted face; (**b**) right side of the cut on the impacted face; (**c**) left side of the cut on the back face of the last layer; (**d**) right side of the cut on the back face of the last layer.

Figure 16 presents a very low magnification (×29 or ×28) for a stab cut in a panel of 16 layers of Twaron^®^ CT 736 CMP (thickness of 8.44 mm). The shape of the cut is dependent on the blade position against the yarns. It is difficult to differentiate, at least in this case, the qualitative damage aspect of a particular layer, depending on its position in the panel. The protective coating of the aramid fabric is cracked.

**Figure 17:**
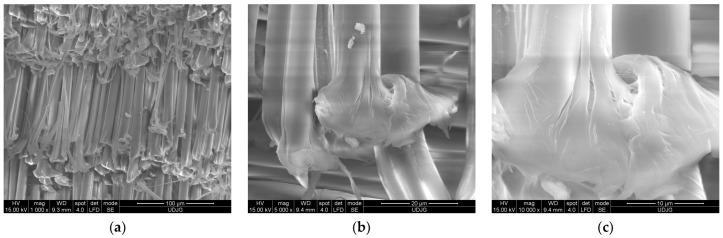
Fracture aspects of aramid fibers cut by knife S1 [66], at 24 J, from a panel made of 16 layers of Twaron^®^ CT736, back view of layer 2: (**a**) ×1000; (**b**) ×5000; (**c**) ×10,000.

Figure 17 presents broken aramid fibers from fabric CT736 CMP [65] used in a panel tested in stabbing with a knife S1 [80], with an energy of 24 J: (a) a magnification of ×1000 points out many fibers broken by shear; (b) fibrillation is visible on both fibers, noticing that fibrils are different sizes; the fiber in the left side is broken by a straight shear cut (a smaller “nail flower”), but the fiber in the right side has a more full-blown end, suggesting a compression between blade and adjacent fibers; (c) this detail of the full-blown fiber end, at higher magnification (×10,000), reveals cross-linking nanofibrils and local splits between fibrils. On layer 2 (from a panel of 16 layers), the fiber ends have smaller nail flowers, meaning the impact energy of the blade is reduced. The pressing of the blade on fibers split them in fibrils.

**Figure 18:**
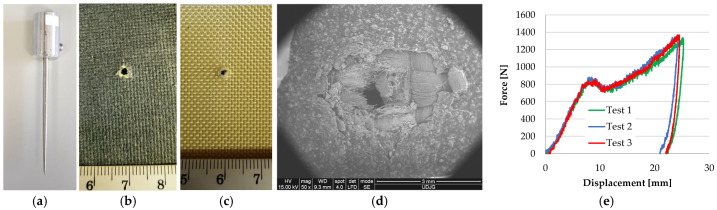
Photos of the panel sample made of 16 layers of fabric SRM509 (Teijin Aramid): (**a**) the spike, with geometry as in [80], (**b**) impacted face of the panel; (**c**) back of the same panel; (**d**) low magnification (×50) of the orifice produced by the spike on the panel face; (**e**) force—displacement curves for three repeated tests, with spike attack having an impact energy of 24 J.

A spike could be more aggressive to the fabrics due to its smaller and sharper shape. Figure 18 presents the sample from which the SEM images were taken. When a spike tries to break through a panel made of stratified fabrics, its action differs as compared to a knife, mainly due to its conical shape and small dimension in cross section. The SEM image in Figure 18d could be considered typical for this test (spike, strike energy 24 J) as the curves force–displacement are very close one to another (Figure 18e).

**Figure 19:**
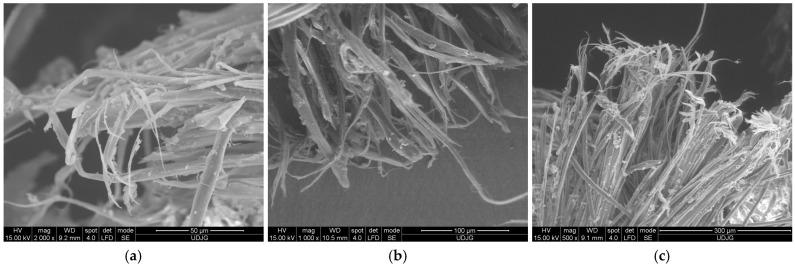
SEM images of the broken fibers from a panel made of 16 layers of SRM509, under spike attack (with geometry as in [66], with an energy of 24 J): (**a**) layer 1, face view: fiber fibrillation at broken end, (**b**) layer 2, face view: fibrillation of several fibers, the fibrils’ length suggests successive breakage, (**c**) layer 2, back view: fibrillation of fibers, the ends of broken fibrils are bent, probably because of spike passing through the orifice.

Figure 19 presents fibers after facing a spike attack. The sharp pointed rod attacks less yarns and, implicitly, less fibers will be cut [81]. Till this fracture occurs, the fibers are elongated. The last layers are easier to be displaced and, thus, the tensile stress is one of the most important causes of fiber breaking. Of course, before a break, the fiber fibrillates and becomes thinner.

**Figure 20:**
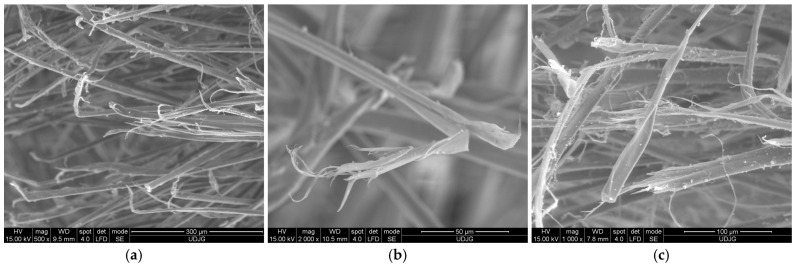
Aspects of failed fibers from panels made of fabrics Twaron^®^ T730, layer 1: (**a**) projectile 9 mm FMJ, v_0_ = 371 m/s (from a panel of 5 layers); (**b**) projectile 9 mm FMJ, v_0_ = 371 m/s (from a panel of 15 layers); (**c**) projectile .357 SIG, v_0_ = 448 m/s (from a panel of 15 layers).

Figure 20 presents aramid fibers broken by traction. They are, statistically speaking, less numerous as compared to fiber cut by shearing. The fiber is elongated due to its position to the knife blade or spike. Usually, the weapon tip presses the fiber, not enough to cut, but it will be tensioned near the compression zone till it breaks. This failure is recognized by the necking of the fiber, the splitting of fibrils that will break successively, depending on their thickness. This fracture mechanism is statistically more present after ballistic hits.

In stabbing or puncture processes, flattening and fibrillation of fibers increase resistance by spreading the applied load, making it harder for sharp objects to penetrate.

**Figure 21:**
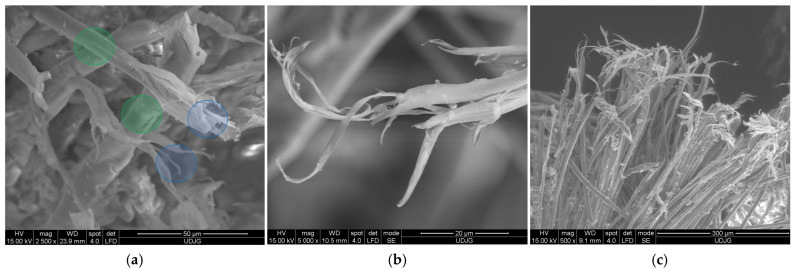
Fibrillation of aramid fibers: (**a**) fabric Twaron^®^ LFT SB1 in PVB matrix, ballistic test with projectile 9 mm FMJ, (**b**) Twaron^®^ T730, panel of 15 layers, ballistic test with projectile 9 mm FMJ, v_0_ = 371 m/s, (**c**) broken fibers from layer 2 (back view) from a panel with 16 layers of fabric Twaron^®^ SRM 509, after a spike test at 24 J. The blue circles point out the fibrillation at the end of the fibers, and the green circles indicate local fibrillation on the fibers, not at their ends.

Figure 21 presents fibrillation of aramid fibers under different test conditions:

(a) fibers in the front view of the orifice produced by a projectile of 9 mm FMJ (v_0_~400 m/s) in a panel made of 32 layers of Twaron^®^ LFT SB1 (a biaxial fabric, with 200 g/m^2^, thickness 0.3 mm), in a PVB matrix [43]. The blue circles point out the fibrillation at the end of the fiber, meaning stress was enough to split the fibrils and to tear them off (tensile loading) until break. On the same SEM image, the green circles indicate local fibrillation induced by high tensile stress,
(b) the fibrillated end of a fiber broken by the projectile attack on the panel made of 15 layers of Twaron^®^ T730; the curly aspect is due to fibril elongation during bullet contact and then the fibrils have an elastic component that allows for changing their shape,
(c) under a spike action (with the geometry from [80]), main yarn has fibers cut due to tensile loading and the fiber ends are fibrillated on different length, meaning the individual load on fibers could have large value range.

Fibrillated fibers of one layer entangle with fibers in adjacent layers, enhancing the overall structural integrity and resistance. The intensity of fibrillation could be different for different layers of the panel as the impact energy decreases from the first to the last layer, and the presence of layers above and under the layer of interest influences the failure intensity and aspect.

**Figure 22:**
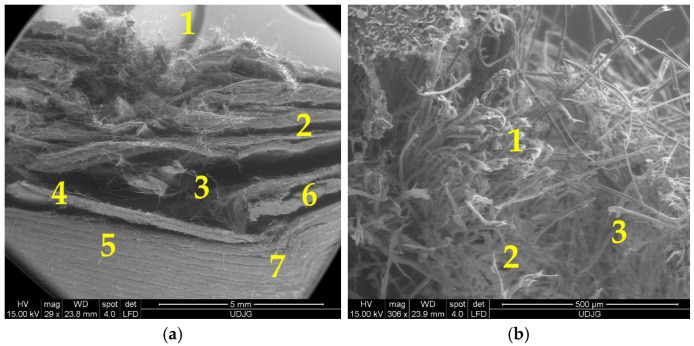
Small magnifications on a cross section of the orifice produced by a 9 mm FMJ projectile on a panel made of 32 layers of Twaron^®^ LFT SB1, as tested in [70]: (**a**) ×29; (**b**) ×306.

In a low magnification SEM image (×29), one may identify failure mechanisms at macro-scale (Figure 22a), noticing the damage layer positions, the penetration depth and the orifice shape, produced by projectiles or sharp weapons: 1—first layers: yarn and fibers have different failure mechanisms, 2—zone with delamination above the zone where the projectile was arrested (stopped); it could be noticed that delamination occurs between fabrics and the two sub-layers remain still together, meaning the technology of sticking these two layers, with yarn arrangement (0°, 90°), has a particular role in energy absorption, 3—this void was generated by the deformed projectile because the layer under it was not broken and the projectile was obliged to expand laterally (the projectile was extracted), 4—last broken layer: it could be noticed the different orientation of the two sublayers, 5—composite zone with no delamination under the projectile, 6—severe delamination due to projectile deformation, the shape and asymmetry of layer separations suggest a very asymmetric deformation of the projectile, 7—a zone with small local delaminations and layers’ deformation, suggesting that a fragment or a sharp edge of the projectile generates this more severe local damage, but without layer breakage. In Figure 22b, a “closer look” (magnification of ×306) enables differentiation of the fiber failures. In zone 1, a bundle of aramid fibers is cut (sheared), zones 2 and 3 point out fibrillation on the broken end of aramid fibers and necking caused by traction.

**Figure 23:**
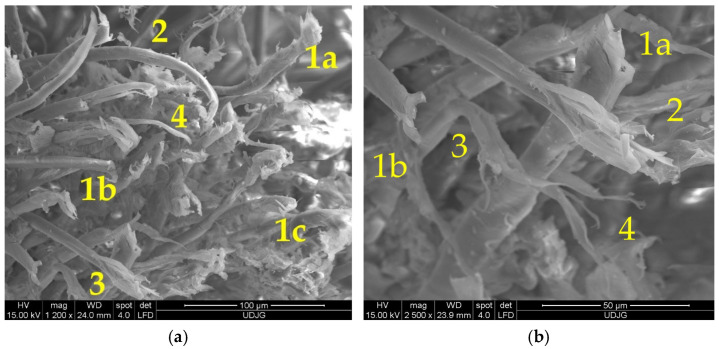
Greater magnifications of the same orifice in Figure 21a, from the first layer of a panel made of Twaron^®^ LFT SB1 layers in a matrix of PVB, after being hit with a 9 mm FMJ bullet (v_0_~400 m/s): (**a**) 1a, 1b and 1c—fibers broken by shear; 2—fiber with a small closed fibrillation; 3—m fiber with a combination of damages, fibrillation, shear and crush; 4—fiber with necking (×1200); (**b**) 1a and 1b—fibers cut by shearing, 2—a broken end of a fiber, split in several fibrils, 3—a fiber with local bending and fibrillation, 4—the broken ends of fibrils from fiber 3 (×2500).

Analysis of a stratified composite made of 32 layers of Twaron^®^ LFT SB1, after being hit with a projectile 9 mm FMJ, is given in Figure 23: (a) 1a, 1b and 1c are fibers broken by shear; fiber 2 has a small closed fibrillation, but the fiber under this one exhibits a fibrillation at its broken end; fiber 3 presents a combination of damages, including fibrillation, shear and crush; fiber 4 presents a visible necking. In a small square area with a side of around 200 μm, more than four types of failure mechanisms could be identified. At magnification ×2500, Figure 23b points out similar failure mechanisms: 1a and 1b are fibers cut by shearing, 2—a broken end of a fiber that was split in several fibrils, each one with a different shape, obtained, very probably, by local shear and tensile load and bending; 3—a fiber with local bending and fibrillation, and 4—the broken ends of fibrils from fiber 3: two are very thinned, the other is twisted, locally flattened.

**Figure 24:**
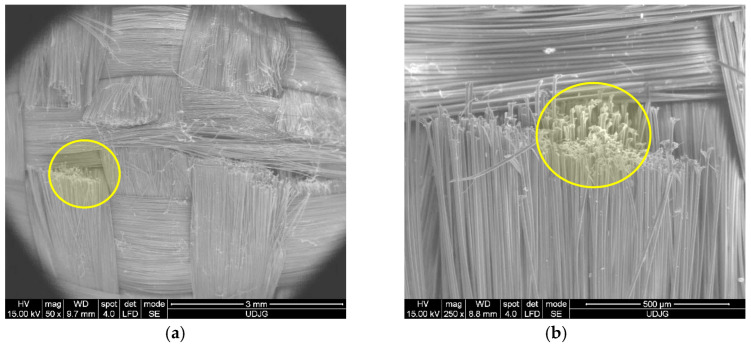

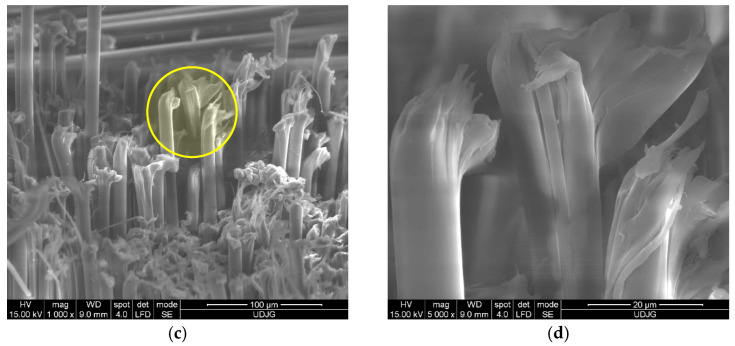
Typical aspect of fibers cut on a panel made of 16 layers of Twaron^®^ CT736 CMP, stabbed with knife S1 [80], impact energy 24 J; layer 1 from the panel, back view: (**a**) ×50; (**b**) ×250; (**c**) ×1000; (**d**) ×5000. (The yellow circle marks the zone of interest, magnified in the next SEM image.)

Figure 24 presents an investigation of a knife attack (geometry S1, as in [80]). The circles indicate the zone with increased magnification in the next image. The SEM images in Figure 24 are described as follows: (a) left end of the cut produced on the panel, front view of the first layer; one may notice that the woven fabric has transversely cut the yarns (those that were positioned perpendicular to the blade width) and the yarns that are almost parallel to the knife blade, partially cut but disorganized, when the weapon laterally pushes the yarns, without cutting them; (b) detail with a yarn with fibers cut almost in the same plane (fibers were attacked by the sharp blade), (c) detail of a bunch of fibers broken by shear action of the blade, typical nail flowers are seen at the broken fiber ends; (d) a higher magnification (×5000) points out details of the broken ends of several fibers: the compressive action of the blade (before shearing the fibers) produces fibrillation but on a short length of the fibers and these fibrils are successively sheared, tips of the fibrils being then bent or/and flattened due to the sliding of the blade along them.

## 6. Using SEM for Identifying the Failure Mechanisms of Aramid Fibers

In the chapter “High Resolution Imaging”, Goldstein J. I. et al. [63] present methods to improve imaging at high magnification, referring to polymers also.

The following SEM images illustrate and explain the aramid fibers’ failures, as obtained from three tests: ballistic impact, stabbing and puncture.

Issues concerning good quality SEM images for fibers, especially aramid fibers, include sample preparation, imaging parameters, space orientation of fiber(s) and their topography, measurement and interpretation. When dealing with polymeric materials, electron charging is a critical issue. Aramid fibers are inherently non-conductive, making them prone to accumulating electrons when exposed to the electron beam. This charging can cause several issues:

- distorted images as charging creates bright spots or dark patches that obscure surface details,
- electron beam deflection, leading to blurred or misaligned images,
- surface damage of the micro-areas under investigation because prolonged charging may alter the surface morphology, especially in delicate fibers.

How to manage these unwanted effects? A solution is to apply a very conductive coating, like gold, platinum or carbon, which allows electrons to dissipate, preventing charge buildup. It is recommended that lower beam energy is used because it reduces the amount of charge introduced into the sample. An accelerating voltage of 1–5 kV is helpful, depending on the fiber’s thickness and required resolution. The sample mounting on conductive adhesive tape or a metallic substrate (e.g., aluminum stubs) ensures good electrical contact between the sample and the SEM stage. Fibers embedded in resin ensure the resin block has a conductive path to the stage. The localized charging could also be reduced by avoiding excessive exposure of the same area, reducing dwell time per pixel to minimize charge buildup.

Often, a combination of these techniques could produce optimal results. For instance, for the SEM images in this study, the fibers were coated with a thin layer of gold, they were investigated in low-vacuum mode, with lower energy beam and reduced dwell time.

Stab and spike failure mechanisms are generated by lower velocities (around 4 m/s) [46,81,82], and a concentrated force that seeks to exploit the gaps between fibers [83]:

- fiber displacement: blades and spikes may force fibers aside rather than breaking them, slipping through the weave structure;
- shear failure: sharp blades cut through fibers, depending on the blade’s sharpness, rigidity and position to the yarns;
- in spike protection, shear forces generated from narrow, pointed objects can cause fibers to fail by shearing or traction across their cross sections.
- microbuckling: the compressive forces in the fiber structure from a stab can lead to localized buckling, reducing load-bearing capability.

Design solutions for mitigating fiber failure mechanisms for all three analyzed cases (ballistic, stabbing or puncture), at micro-level, could be:

- use of tightly woven structures: reducing freedom of individual yarns and fibers limits localized deformation and necking and forcing neighboring fibers to take over some of the load;
- use of panel hybridization by introducing different materials, in an order to improve the performances of the protective system: combining aramid fibers with materials that have higher elongation-to-break ratios (like ultra-high-molecular-weight polyethylene) can distribute the strain more evenly; adding more rigid fabrics (carbon fibers) in front of the panel could be efficient for stabbing and spike attacks;
- applying coatings and treatments for fibers: surface treatments can enhance fiber toughness and delay necking and even break; the coating could improve adhesion between fibers and matrix, delaying debonding and delamination, thus, keeping together fibers and layers.

When an aramid fiber breaks under tensile stress or impact, the fracture is often a net cross section. Instead, the ends of broken fibers become flattened, a phenomenon linked to the structure of the polymer chains.

During high-stress loading, the region near the fiber break experiences severe deformation. The polymer chains align and stretch, causing the ends to flatten. But SEM images reveal also local deformation (necking, flattening or composed deformations) on the fiber, far from the fiber’s broken end. This is due to flows in structural architecture of the fiber, variation in crystallinity, etc.

Necking and flattening are ways in which the material absorbs and dissipates energy, especially during ballistic impacts. This deformation helps to blunt the fracture and prevent immediate propagation.

The presence of flattened ends suggests ductile behavior before failure, indicating that the material absorbed energy in this way, too. Flattening can slightly increase the load-bearing capacity after initial failure, as it may slow down further crack propagation. Analysis of broken fibers and flattened ends can help forensic experts understand the nature and severity of impacts.

Local bending is another key factor influencing the performance of aramid fibers, especially under impact or penetration forces. It refers to the deformation of individual fibers or yarns within a fabric when subjected to a concentrated load. This bending can occur without the fiber completely breaking, or it may lead to failure if the bending stress exceeds the fiber’s strength. In woven fabrics, local bending due to crossing yarns could decrease the overall resistance as compared to unidirectional or multiaxial fabrics.

When a bullet, blade or spike impacts a vest, the load is applied to a small area, causing fibers to bend sharply near the contact zone, especially if the panel has higher elasticity (or no matrix). The bending induces high localized stress on the outer curve of the fiber, where tensile forces are higher. Compression occurs on the inner curve. If the bending stress exceeds the fiber’s strength, cracks may initiate on the tensile side, leading to fracture. In laminated structures, local bending of fibers can also shear or crack the matrix, locally weakening the structure.

There are factors influencing fiber local bending. A smaller fiber diameter offers flexibility, but it may have lower resistance to bend-induced stress, especially when the matrix has already been detached. Weave architecture produces local bending stress and it is influenced by how tightly the fibers are interlaced. Loose weaves allow more bending but may be less effective at distributing loads and opposing the penetrating body. High-velocity impacts cause rapid, intense bending, often leading to brittle fractures before significant deformation. Bending is more likely in fibers oriented at an angle to the impact force. But local bending is also due to the forced advance of the projectile against already broken fibers.

Implications in protective systems could be taken into account, depending on the type of protection:

- for ballistic protection: local bending helps distribute the impact energy across a wider area, reducing the likelihood of fiber fracture; in some cases, fibers can withstand bending without breaking, enhancing the material’s overall toughness; in multi-layer vests, local bending in one layer can transfer the load to adjacent layers, improving energy absorption;
- for stab and spike protection: local bending can deflect or blunt the point of a spike or blade, preventing penetration; excessive bending can displace fibers, creating pathways for penetration if not properly managed; in compression zones during bending, fibers can experience microbuckling, leading to localized failure.

Fiber bending can initiate fibrillation, absorbing energy and delay catastrophic failure. In composites, local bending stresses can cause layers to separate, reducing the material’s integrity.

Understanding local bending and its effects is crucial for optimizing the design and performance of protective body armor, ensuring better resistance to both ballistic and stab threats.

The relatively new challenge for the protective systems is to face more than one threat. Thus, a body armor is designed and tested for ballistic, but also bladed weapons and this study helps select the solution by understanding the behavior of aramid fibers under such severe impacts, at micro-level.

**Figure 25:**
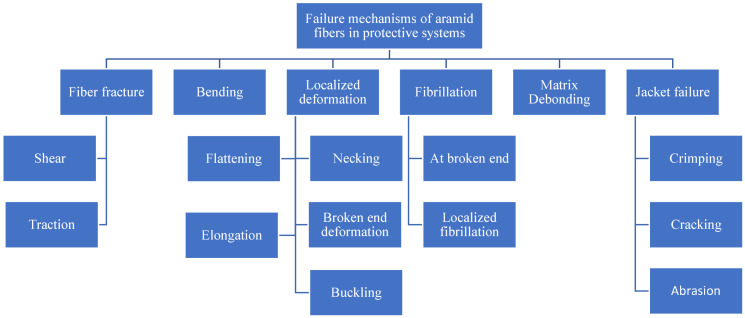
Classification of mechanical failures of aramid fibers used for protective systems.

Figure 25 presents a suggestive classification of failure mechanisms of aramid fibers in protective systems, based on SEM images previously discussed.

The three cases of damaging body armors (ballistic, knife and spike hits) are discussed here, and the authors present resemblances and differences in aramid fibers’ failure which a protective system could face and the response of the involved materials, analyzed at micro-scale, which could promote original ideas for improving the design.

Aramid fibers could fail due to mechanical, thermal and chemical causes.

Thermal failures include decomposition, oxidative degradation, glass transition and melting behavior and synergic damage by the environment (including exposure to UV, moisture and environmental composition).

Designers of body armors have key strategies for mitigating these failure mechanisms. For all three cases here analyzed, stratified and hybrid materials could offer better behaviors. Using multiple layers of different fabrics or combining aramid with other materials (like ceramics, carbon fiber fabrics) could improve performance. This performance must be evidenced under actual test conditions, even if the first solution is given by simulation or/and a recent documentation. Research of hybrid composites and 3D architecture for different individual protection systems, containing aramid fibers are reported in [82,83,84,85], for bullet-proof vests and for stabbing and spike attacks in [86,87,88,89,90,91].

Weave density optimization has a straightforward direction for these loading cases: tight weaves resist ballistic and stab attacks better by reducing fiber displacement.

Protective coatings enhance resistance to UV, moisture and abrasion. There are coatings that improve the stabbing resistance, but their behavior in combined protection (both ballistic and knife/spike attacks) could be controversial.

**Figure 26:**
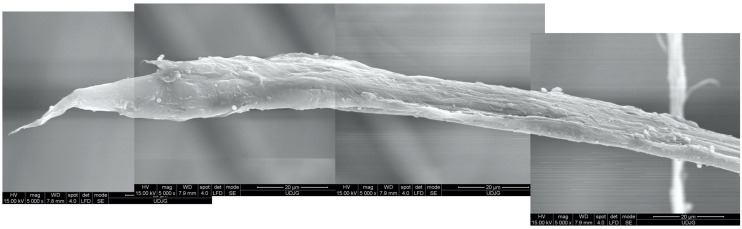
Collated SEM images of a fiber from layer 1, from a panel with 5 layers of Twaron^®^ T730 WRT, tested with a projectile .357 SIG, v_0_ = 448 m/s (total penetration).

As aramid fibers could present several failure mechanisms on their lengths, the SEM images could be collated in order to analyze a complex aspect. For instance, Figure 26 is obtained from four SEM images carried out at the same magnification (×5000) and under the same microscope parameters. The fiber is broken by traction, but it exhibits an end necking. The fiber core is visibly made of different size fibrils. The fiber jacket has remained only partially attached to the core, but a local split from the core is also present. It is very probable that other fibrils were detached from the upper side of the fiber.

## 7. Conclusions

Scanning electron microscopy (SEM) is a powerful tool for investigating the failure mechanisms of aramid fibers. By providing high-resolution imaging and detailed surface characterization, SEM reveals critical insights into the microstructural changes and damage patterns that lead to fiber failure.

Based on SEM studies, presented in this paper, the following conclusions can be drawn.

SEM analysis enables the identification of specific failure modes in aramid fibers and provides qualitative arguments to validate theoretical models of fiber failure. These models enable us to reduce the financial effort necessary for actual tests.

Microscopic evidence of damage patterns can be compared with predictions from experimental stress–strain relationship or models.

Insights from SEM studies guide improvements in strategies for aramid fiber durability and their applications and for mitigating the damages under severe loading, characteristic for ballistic impact, stabbing and puncture.

Identifying specific degradation pathways (e.g., oxidative vs. hydrolytic) helps tailor protective coatings or environmental controls.

Observations of damaged areas could improve fiber fabrication, fabric architecture and design of the final product, with enhanced performance.

SEM provides surface-level information, which must be corroborated with complementary techniques (e.g., spectroscopy or mechanical testing) for comprehensive and reliable analysis.

SEM studies of aramid fiber failure mechanisms are invaluable for understanding how microstructural issues lead to macroscopic failure. These insights enable the optimization of fiber design, processing and application, ensuring better performance and longer service life in demanding applications as those discussed in this paper, for individual protection systems.

## Figures and Tables

**Table 1:** Step-by-step guide for a SEM investigation on aramid fibers.

Selection of fibers or composite sample and handling	Be aware of the maximum dimensions allowed for investigation in the microscope enclosure. Select representative fibers or slices from the entire panel. Avoid contamination and clean samples in order to not affect the functioning of the vacuum enclosure. Use clean tools and wear gloves to prevent oils or dirt from affecting samples. Use an air jet with adequate velocity and flow to remove fragments of fibers that can easily be detached from the sample.
Cutting and mounting the samples with fibers	Cut to size. Use a sharp blade or micro-scissors to cut fibers to a manageable length (typically 5–10 mm). Mounting. Attach single fibers to an SEM stub, using conductive carbon tape or conductive adhesive. Ensure the fibers are securely fixed and extend horizontally or vertically for clear views. Alignment. Orient fibers so their ends or surfaces of interest are facing the electron beam.
Conductive coating	It is optional, but recommended, because aramid fibers are non-conductive, so a thin conductive coating helps prevent charging effects, improving image quality. Also, if the fibers are in a polymeric matrix, the coating also helps show the matrix. The coating could be made of gold, platinum or carbon. Place the sample in a sputter coater or vacuum evaporator. Apply a thin uniform layer (5–20 nm) to avoid obscuring fine surface details.
Vacuum treatment	Ensure the sample is dry and free of contaminants. Some fibers might need pre-drying in a desiccator to avoid outgassing during SEM analysis.
Setting SEM parameters	Low accelerating voltage. Use 5–10 kV to reduce damage to the fibers. Low beam current. Prevents charging and heat damage. High vacuum mode. Enhances resolution and contrast.
Imaging	Begin with a low magnification to locate areas of interest, then increase magnification. Adjust focus, contrast and working distance to optimize image quality.
Safety considerations	Handle materials safely, as aramid fibers can be brittle and create fine particles. Ensure proper ventilation when dealing with any coatings or adhesives.

**Table 2:** Trade names and specifications for fabrics or prepegs used in this SEM analysis [64,65].

Fabric or Prepeg Trade Name	Specifications
LFT SB1	2 layers woven fabric + 3 layers thermoplastic film, arranged in 0° and 90° orientations, surface density 430 g/m^2^
LFT SB1plus	4 layers woven fabric + 5 layers thermoplastic film, arranged in 0°, 90°, +45° and −45° orientations, surface density 430 g/m^2^
T730 WRT	930 dtex f 1000, fabric weave—plain, surface density 260 g/m^2^
SRM 509	for the fabric: base yarn Twaron^®^, 930 dtex f 1000, fabric weave—plain, with ends 105 ± 2/10 cm and picks 105 ± 2/10 cm, surface density 200 g/m^2^; for the coating with silicon carbide: abrasive mass—127 g/m^2^, total mass of the prepeg (fabric + coating) 426 g/m^2^
CT 736 CMP	Twaron^®^ 2000/200, 1680 dtex f 1000, fabric weave—plain, surface density 413 g/m^2^; for the coating with silicon carbide: abrasive mass—min 95 g/m^2^, total mass of the prepeg (fabric + coating) 413 g/m^2^, thickness 0.62 mm

## Data Availability

The original contributions presented in this study are included in the article. Further inquiries can be directed to the corresponding authors.
